# Teriparatide for Guided Bone Regeneration in Craniomaxillofacial Defects: A Systematic Review of Preclinical Studies

**DOI:** 10.3390/cimb47080582

**Published:** 2025-07-23

**Authors:** Jessika Dethlefs Canto, Carlos Fernando Mourão, Vittorio Moraschini, Rafael da Silva Bonato, Suelen Cristina Sartoretto, Monica Diuana Calasans-Maia, José Mauro Granjeiro, Rafael Seabra Louro

**Affiliations:** 1 Department of Oral and Maxillofacial Surgery, School of Dentistry, Valparaíso University, Valparaiso 2360004, Chile; jessika.dethlefs@uv.cl; 2 Department of Oral Surgery, School of Dentistry, Fluminense Federal University, Niteroi 24033-900, Brazil; vittmf@gmail.com (V.M.); rafaelbonato@yahoo.com.br (R.d.S.B.); susartoretto@hotmail.com (S.C.S.); monicacalasansmaia@gmail.com (M.D.C.-M.); drrafaelseabra@gmail.com (R.S.L.); 3 Department of Basic and Clinical Translational Sciences, School of Dentistry, Tufts University, Boston, MA 02111, USA; 4 Department of Odontotechnic, School of Dentistry, Fluminense Federal University, Niteroi 24033-900, Brazil; jmgranjeiro@id.uff.br

**Keywords:** teriparatide, bone regeneration, guided bone regeneration, biomaterials, alveolar bone defects, craniomaxillofacial, systematic review

## Abstract

This systematic review aimed to evaluate the effectiveness of teriparatide (TP) in guided bone regeneration (GBR). An electronic search without language or date restrictions was performed in PubMed, Web of Science, Scopus, Scielo, and gray literature for articles published until June 2025. Inclusion criteria considered studies evaluating the effect of TP on bone regeneration, analyzed using SYRCLE’s Risk of Bias tool. Twenty-four preclinical studies were included, covering diverse craniofacial models (mandibular, calvarial, extraction sockets, sinus augmentation, distraction osteogenesis, segmental defects) and employing systemic or local TP administration. Teriparatide consistently enhanced osteogenesis, graft integration, angiogenesis, and mineralization, with potentiated effects when combined with various biomaterials, including polyethylene glycol (PEG), hydroxyapatite/tricalcium phosphate (HA/TCP), biphasic calcium phosphate (BCP), octacalcium phosphate collagen (OCP/Col), enamel matrix derivatives (EMDs), autografts, allografts, xenografts (Bio-Oss), strontium ranelate, and bioactive glass. Critically, most studies presented a moderate-to-high risk of bias, with insufficient randomization, allocation concealment, and blinding, which limited the internal validity of the findings. TP shows promising osteoanabolic potential in guided bone regeneration, enhancing bone formation, angiogenesis, and scaffold integration across preclinical models. Nonetheless, its translation to clinical practice requires well-designed human randomized controlled trials to define optimal dosing strategies, long-term safety, and its role in oral and craniomaxillofacial surgical applications.

## 1. Introduction

The integrity of the alveolar and craniomaxillofacial bones may be affected by factors such as tooth loss, infections, pathological lesions, trauma, congenital deformities, and systemic conditions [1,2]. Guided bone regeneration (GBR) is a widely employed technique in oral implantology to restore bone defects and ensure an adequate receptor site for dental implants [3]. This approach, based on the use of physical barriers to separate regenerating bone from soft tissues, has demonstrated efficacy in various scenarios, particularly in peri-implant defects and post-extraction sockets. However, the success of this technique can be influenced by systemic and local factors, as well as by the type of material [4].

Bone defect regeneration remains a major challenge in oral and maxillofacial surgery, with autogenous, allogenic, xenogenic, and synthetic grafts commonly employed. However, augmentation material does not generally prevent the resorption process and reduces it on the buccal wall [5]. Many different materials and pharmacological agents are used with graft materials; however, autogenous bone grafting remains the gold standard because of its osteogenic, osteoinductive, and osteoconductive properties. The disadvantages of autografts include a second surgical site and associated donor-site morbidity. Given these disadvantages, many researchers are focused on testing the efficacy of different materials to achieve optimum results and long-term success. The most used biological mediators include bone morphogenetic proteins, platelet-derived growth factors, and parathyroid hormone. These materials have been shown to have regenerative potential in numerous preclinical and clinical studies [6,7,8,9].

In recent years, teriparatide (TP), a recombinant human protein comprising the first 34 amino acid fragments of parathyroid hormone (PTH 1–34 analog), has become popular in the treatment of osteoporosis and delays the healing of skeletal fractures due to its anabolic effect, increasing bone formation [10,11]. Teriparatide acts directly in bone formation by increasing the number and activity of osteoblasts, and indirectly through increasing renal tubular reabsorption and intestinal absorption of calcium. The bone anabolic effect of teriparatide extends to long bones, and its function in the post-extraction alveolar repair process has been proven to increase bone formation [1]. Understanding the effect of teriparatide on increasing bone density in alveolar bone healing will allow us to determine protocols to treat bone defects. There are few comprehensive reviews on the use and efficacy of teriparatide for alveolar bone regeneration, such as medication-related osteonecrosis of the jaw (MRONJ), chronic periodontitis, and implant osseointegration [12]. However, those studies reported limited and low-quality evidence and did not focus on a specific regenerative strategy. Moreover, authors emphasized that more studies were still necessary to obtain significant conclusions.

Teriparatide has shown potential to enhance bone regeneration when combined with graft materials, increasing mineralization and bone formation, as evidenced by the preclinical studies reviewed. This systematic review evaluates how the interaction between biomimetic materials and teriparatide can inform the future design of ceramics for healthcare applications, contributing to innovations in regenerative dentistry and aligning with the focus on bio-inspired materials. Therefore, this updated systematic review aims to provide a comprehensive assessment of the impact of teriparatide on GBR outcomes, addressing the quantity and quality of regenerated bone in preclinical models.

## 2. Materials and Methods

### 2.1. Protocol and Registration

The protocol of the present systematic review was based on the framework of The Joana Briggs Institute and is available on the Open Science Framework platform (https://osf.io/xwsn7/, accessed on 15 July 2025). The reporting was based on the PRISMA 2020 explanation and elaboration: updated guidance and exemplars for reporting systematic reviews [13].

### 2.2. Focus Question

“In preclinical models of alveolar and craniomaxillofacial bone defects undergoing GBR, does the administration of teriparatide result in a significant improvement in bone formation, compared to standard GBR protocols without teriparatide?”

### 2.3. Search Strategy

A systematic search was performed by two independent authors (J.D.C. and C.F.M.) in PubMed, Web of Science, Scopus, and Scielo databases. The Open Gray database was used to check gray literature and prevent selection bias. The Research Gate database was used for studies for which full versions were not available online. The terms “Teriparatide”, “Alveolar Process”, “Craniomaxillofacial”, and “Bone Regeneration” were combined using the Boolean operators AND and OR to create the search strategies, which are presented in Table 1.

### 2.4. Eligibility Criteria

Inclusion criteria were based on the PICOS strategy as described below [14].

- Population (P): Bone defects in the oral cavity and craniomaxillofacial bones treated with guided bone regeneration.
- Intervention (I): Use of teriparatide in guided bone regeneration.
- Comparison (C): Control groups receiving placebo, saline, or GBR without teriparatide.
- Outcome (O): Quantity and quality of regenerated bone, bone density, bone formation rates, and graft integration.
- Study design (S): Experimental and in vivo studies.

The eligibility criteria address the outcome of ‘teriparatide’ and ‘bone regeneration’. Manuscripts, books, letters to the editor, narrative reviews, commentaries, case series, and case reports were excluded. Additionally, recommendations, in vitro studies, and expert statements were excluded. No language and date restrictions were applied. Regarding studies for which full versions were not available online, two attempts were made through the platform ResearchGate, with a predefined deadline of five days for receiving a response. After this period, studies that were not obtained were excluded.

### 2.5. Study Selection

Two independent authors (J.D.C. and C.F.M.) analyzed the articles, discarding in the first instance those whose title and/or abstract did not meet the eligibility criteria. The selected articles were imported into the systematic review analysis platform, Rayyan (https://www.rayyan.ai, accessed on 15 July 2025), where their titles, abstracts, access, and duplicates were evaluated and classified as included or excluded according to the aim of the present review. Subsequently, a full-text review of each selected article was carried out, and those that met the eligibility criteria were included. Discrepancies were resolved by consensus discussion.

### 2.6. Data Extraction

A predesigned data extraction form was used, and the information on each article was compiled and organized in a spreadsheet. The following key information was collected: (1) bibliographic information, including author and publication year; (2) study design; (3) sample size; (4) bone defect model; (5) outcome measure; (6) teriparatide dose; (7) control group; (8) mean bone formation (TP group); (9) mean bone formation (control group); and (10) key findings. One reviewer (J.D.C.) collected the data, and another (C.F.M.) checked all the data.

### 2.7. Quality Assessment

Two reviewing authors (J.D.C. and C.F.M.) analyzed the quality assessment to ensure that studies were reported in enough detail to evaluate their methodological steps and replication of methods and findings in SYRCLE’s Risk of Bias tool [15].

## 3. Results

### 3.1. Study Selection

**Figure 1:**
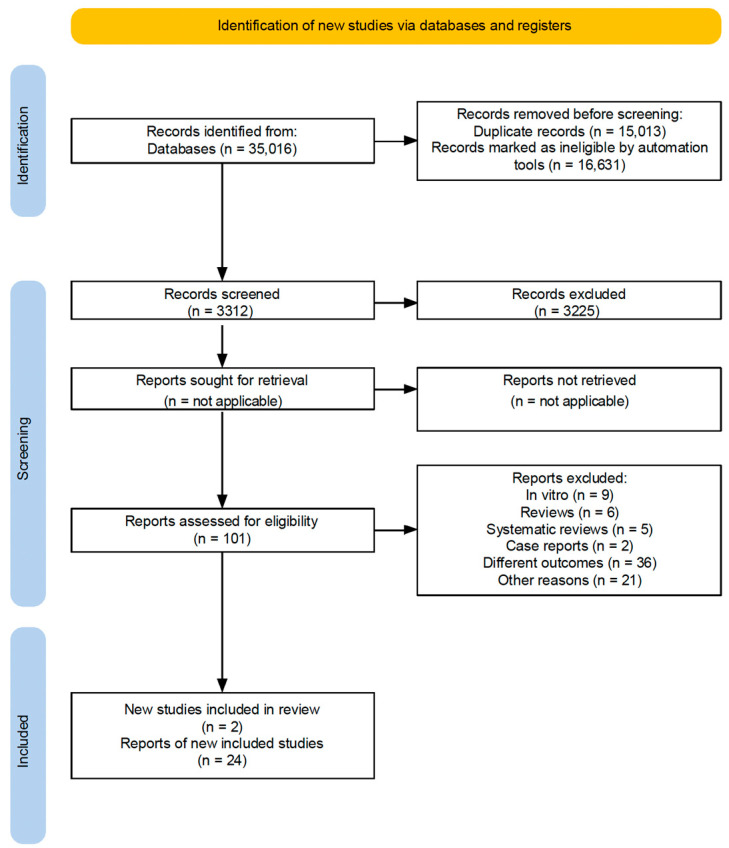
Flowchartof executed search strategy and screening protocol.

The initial electronic search yielded 35,076 records. After duplicates and ineligible records were removed, 3312 records remained for title and abstract screening. Of these, 3225 were excluded, and 101 were advanced for detailed assessment. Among the 101 articles assessed for eligibility, 79 were excluded for various reasons. A supplementary hand search added two additional articles. Thus, 24 studies satisfied the eligibility criteria and were included in this review (Figure 1).

### 3.2. Characteristics of the Included Studies

The study design included 24 experimental studies and a total sample of 1007 specimens. The volume of evidence on this topic has increased significantly in recent years, particularly over the last decade. The number of samples and characteristics of included studies are presented in Table 2 and Table 3, respectively.

### 3.3. Data Extraction

The characteristics of the included studies can be seen in Table 3. The main objective of the reviewed studies was to analyze the ability of teriparatide to stimulate bone formation in critical-sized defects, with a significant body of evidence indicating its potential in several animal models [1,2,16,17,18,19,20,21,22,23,24,25,26,27,28,29,30,31,32,33,34,35,36,37]. Research has focused on the effects of intermittent administration of TP, highlighting its influence on calvarial, mandibular and alveolar bone formation and indicating a notable increase in osteoblastic activity, angiogenesis, and bone formation surface at the tissue level [1,2,16,17,18,19,20,21,22,23,24,25,26,27,28,29,30,31,32,33,34,35,36,37].

A primary finding from this systematic review is the up-regulation of vascular endothelial growth factor (VEGF) and osteogenic markers, including osteocalcin and RANKL/OPG ratio, in teriparatide-treated groups [1,2,34]. This molecular response suggests that teriparatide enhances both angiogenesis and osteoblast differentiation, two fundamental processes in guided bone regeneration (GBR). The addition of teriparatide to xenografts and biomaterials has been associated with an increase in bone mineralization, which suggests an enhanced bone remodeling–substitution process [31,32,37].

However, studies have indicated that intermittent teriparatide may not promote new bone formation in augmented maxillary sinuses in osteoporotic models induced by ovariectomy and glucocorticoids, or even in healthy models [26,30]. Despite this, the integration of mandibular allografts with host bone was shown to be enhanced with intermittent teriparatide administration in pig models [31]. In additional findings, single local doses of teriparatide have demonstrated efficacy in improving allograft integration in rat mandibles [33]. Histological evaluations revealed that teriparatide-treated bone exhibited superior mineralization and reduced porosity, facilitating more stable graft incorporation [1,2,34].

Local administration of TP in biomaterial scaffolds, such as octacalcium phosphate collagen (OCP/Col), has demonstrated significant efficacy in promoting new bone formation in rat calvarial defects. Reported values have exceeded 50%, and the results have shown the formation of well-organized cortical-like bone structures [27]. The dosages and schedules of teriparatide administration appear to be significant, with studies suggesting that the total dose is directly related to the degree of bone regeneration within a set period [24]. A higher total weekly dose administered three times per week has shown superior results compared with daily or weekly regimens, suggesting that the quantity and frequency of TP exposure modulate the regenerative response [24,28].

Additionally, continuous local delivery systems, such as bioactive glass scaffolds functionalized with teriparatide, have shown encouraging results by providing a sustained anabolic stimulus, thereby increasing the amount of newly formed bone even under osteoporotic conditions [37]. It is also important to note that combinations of teriparatide with other pharmacological agents, such as strontium ranelate, have demonstrated synergistic effects in ovariectomized models, resulting in significantly greater bone regeneration than either agent alone and showing enhanced bone quality and implant biocompatibility without eliciting inflammatory responses [28].

### 3.4. Risk of Bias Assessment

**Figure 2:**
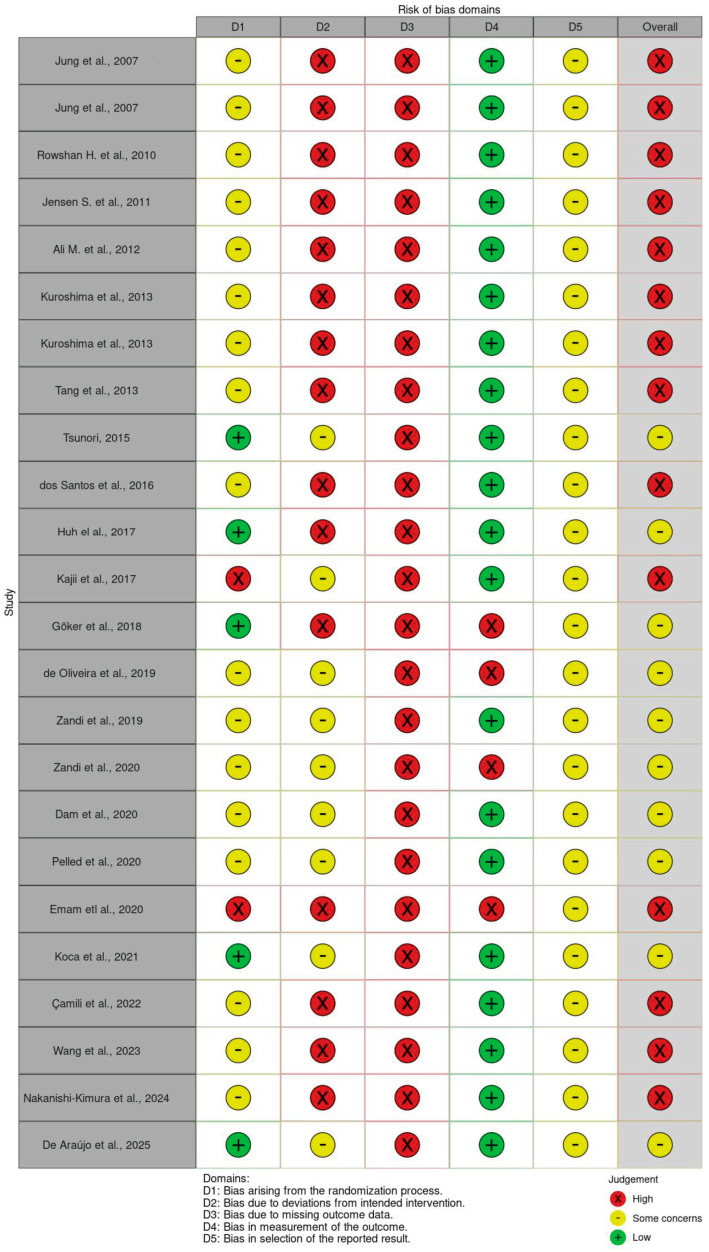

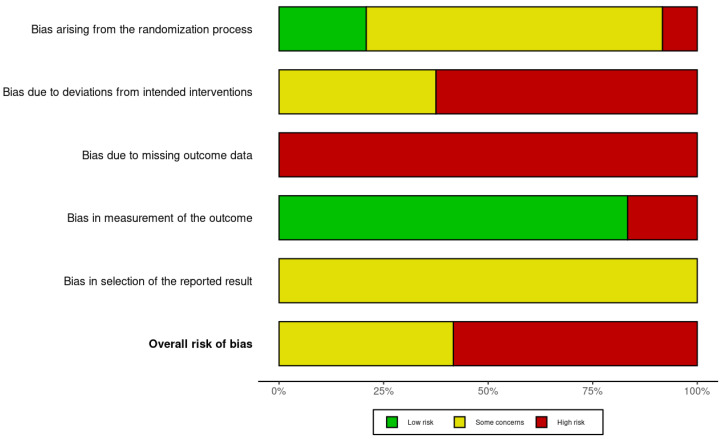
SYRCLE’s risk of bias [1,2,16,17,18,19,20,21,22,23,24,25,26,27,28,29,30,31,32,33,34,35,36,37,38].

The methodological quality of the included preclinical studies identified key limitations in allocation concealment, blinding procedures, and selective outcome reporting, as shown in Figure 2. The results revealed a high prevalence of unclear and high-risk domains, suggesting potential biases that may influence the reliability of reported findings on teriparatide in GBR.

## 4. Discussion

This systematic review aimed to determine whether teriparatide administration enhances bone regeneration and graft integration in preclinical models of guided bone regeneration (GBR). The included studies evaluated teriparatide in various models of alveolar and craniofacial bone defects using systemic and local administration methods. Teriparatide’s anabolic effects mainly occur through stimulation of osteoblast differentiation, suppression of osteoclast activity, and promotion of angiogenesis. These biological mechanisms result in increased trabecular thickness and bone volume, as well as improved mechanical properties. This is supported by the up-regulation of angiogenic (VEGF) and osteogenic markers, including osteocalcin and the RANKL/OPG ratio.

In terms of administration strategies, both systemic intermittent dosing and local single-dose applications have been shown to be effective. Tsunori et al. revealed that systemic regimens increased bone formation by 40.3% to 66.2%. The highest regeneration was achieved with 105 µg/kg administered three times per week, compared to 10.3% in the control [24]. Zandi et al. reported that systemic teriparatide (2 µg/kg/day) combined with autologous grafts significantly enhanced bone formation (52.7%), as compared to grafts alone (38.5%) or untreated defects (18%) [2]. In a segmental mandibular defect model in minipigs, Pelled et al. found that systemic administration of 1.75 µg/kg/day over eight weeks increased bone volume and mechanical strength by 46.3% and 32.3%, respectively, underscoring its regenerative and biomechanical potential [31].

Consistent, dose-dependent improvements in bone regeneration were observed across the included studies when teriparatide was locally applied to diverse biomaterial scaffolds. Kajii et al. demonstrated that octacalcium phosphate collagen (OCP/Col) scaffolds loaded with 0.1–1.0 µg of teriparatide achieved significantly greater bone formation (29.2–38.1%) compared to OCP/Col alone (17.5%), highlighting its osteoinductive synergy [27]. Emam et al. reported that combining teriparatide with Bio-Oss enhanced mineralization, density, and hardness in mandibular xenografts in a porcine model [32]. Koca et al. observed that a single 40 µg local dose significantly improved allograft integration (23.3%) compared to allografts alone (18.4%) or untreated defects (8.3%) [33].

Jung et al. evaluated synthetic polyethylene glycol (PEG) matrices combined with hydroxyapatite/tricalcium phosphate (HA/TCP) in calvarial defects and PEG functionalized with RGD peptides in mandibular defects. Both studies showed that incorporating teriparatide resulted in superior bone regeneration [16,17]. However, Jensen et al. found no additive benefit when teriparatide or enamel matrix derivative (EMD) was combined with PEG and biphasic calcium phosphate (BCP) scaffolds in minipigs [19].

Zandi et al. demonstrated in mandibular models that systemic teriparatide (2 µg/kg/day) significantly improved the healing of autologous iliac grafts compared to graft-only and untreated controls [2]. In ovariectomized rat models, Göker et al. reported synergistic effects when teriparatide was combined with strontium ranelate, outperforming the individual agents in improving bone volume and microarchitecture [28]. De Araújo et al. highlighted the effectiveness of teriparatide-functionalized 45S5 bioactive glass, significantly enhancing bone repair under osteoporotic conditions [37]. However, not all studies demonstrated positive outcomes. Huh et al. found no significant difference in bone formation in healthy rabbit sinus augmentation models after short-term intermittent administration (10.85% vs. 10.45%) [26]. Similarly, enhanced maturation but no significant early volume gain in sinus grafts in ovariectomized rabbits was noted by Dam et al., suggesting that teriparatide’s efficacy may be critically influenced by anatomical site, vascularization, and healing process [30].

A consistent dosing trend emerged across all models. Systemic regimens ranged from 0.5 to 2.5 µg/kg/day, and local applications ranged from 0.1 to 40 µg. Intermediate systemic doses of 2 µg/kg/day and local doses between 20 and 40 µg appeared to be most effective. Higher doses were not universally superior and should be evaluated carefully due to potential adverse effects. Teriparatide’s effects were also increased when combined with scaffolds or grafts, especially under conditions of reduced bone turnover, such as after ovariectomy or orchiectomy.

Despite promising results, several methodological limitations must be acknowledged. The critical risk of bias (RoB) assessment, conducted using the SYRCLE tool, revealed that none of the included studies met all criteria for low risk. Common weaknesses included inadequate reporting of random sequence generation, lack of allocation concealment, absence of random housing, and insufficient blinding of caregivers and outcome assessors. Notably, only three studies (Pelled [31], Koca et al. [33], and Wang et al. [35]) demonstrated partial methodological rigor in key domains. Overall, the majority of studies were rated as having moderate-to-high risk of bias, with incomplete outcome data and selective reporting further undermining internal validity and reproducibility.

Importantly, none of the studies adhered to established preclinical reporting standards such as the ARRIVE or PREPARE guidelines. This limits comparability across studies and hinders translational relevance. Heterogeneity in animal species, defect models, follow-up periods, administration regimens, and outcome measures further complicates drawing firm conclusions and establishing standardized protocols.

Future research should address these gaps by incorporating rigorous randomization, blinding, complete outcome reporting, and standardized methodologies. Additionally, investigating clinically relevant scenarios, such as trauma-induced bone loss, post-resection reconstruction, osteoporotic conditions, and peri-implant regeneration, is essential to validate teriparatide’s translational potential. Furthermore, carefully designed dose-escalation studies and combination therapies are needed to maximize therapeutic efficacy while minimizing adverse effects.

## 5. Conclusions

This systematic review presents strong preclinical evidence supporting the osteoanabolic effects of teriparatide (TP) in guided bone regeneration (GBR) models. TP consistently promotes bone formation, osteoblast differentiation, angiogenesis, and graft integration across various animal studies. These effects are enhanced by different biomaterials, including synthetic matrices (PEG, β-TCP, BCP, HA), OCP/Col, enamel matrix derivatives, autografts, allografts, xenografts (Bio-Oss), and bioactive glass.

Intermediate systemic doses (~2 µg/kg/day) and local doses (20–40 µg) appeared most effective, although higher doses did not consistently produce better results. Anatomical variability was noted, with strong effects in mandibular, calvarial, and extraction socket models, but limited benefits in sinus augmentation. However, most of the included studies showed a moderate-to-high risk of bias, especially in randomization, blinding, and outcome reporting. These limitations restrict the internal validity of the findings.

Further well-designed randomized clinical trials are crucial to identify optimal dosing, delivery methods, and the safety profile of TP for oral and maxillofacial bone regeneration.

## Figures and Tables

**Table 1:** Searchstrategy.

Database	Search Strategy	Results
**Pubmed**	#1 teriparatide (Mesh terms) OR PTH 1–34 (Mesh terms)	379
	#2 alveolar process (Mesh terms)	1161
	#3 bone regeneration (Mesh terms)	1479
	#4 craniomaxillofacial (all fields)	3834
	#1 and #2	5
	#1, #2 and #3	1
	#1, #2, #3 and 4	0
**Web of Science**	#1 teriparatide OR PTH 1-34	113
	#2 alveolar process	2138
	#3 bone regeneration	8743
	#4 craniomaxillofacial	596
	#1 and #2	36
	#1, #2 and #3	35
	#1, #2, #3 and 4	27
**Scopus**	#1 TITLE-ABS-KEY Teriparatide OR PTH AND 1-34	101
	#2 TITLE-ABS-KEY Alveolar AND Process	6986
	#3 TITLE-ABS-KEY Bone AND Regeneration	8675
	#4 craniomaxillofacial	423
	#1 and #2	4
	#1, #2 and #3	0
	#1, #2, #3 and 4	0
**Scielo**	#1 teriparatide	17
	#2 alveolar process	76
	#3 bone regeneration	152
	#4 craniomaxillofacial	30
	#1 and #2	0
	#1, #2 and #3	0
	#1, #2, #3 and 4	0
**Open Grey**		0
**Research Gate**		5
**Total**		35,01 6

**Table 2:** Characteristics of included studies.

Charact Eristics of Included Studies		
Experimental	a *	24
	b **	1007
Publication Year		
Before 2000	a	0
	b	0
2001–2005	a	0
	b	0
2006–2010	a	3
	b	51
2011–2015	a	6
	b	230
2016–2020	a	10
	b	538
2021–2025	a	5
	b	188

* (a) number of studies; ** (b) sample size.

**Table 3:** Data extraction.

Author and Year	Study Design	Sample Size	Bone Defect Model	Outcome Measures	Teriparatide Dose Route of Administration	Control Group	Mean Bone Formation (TP Group)	Mean Bone Formation (Control Group)	Key Findings
Jung et al., 2007 [16]	New Zealand white rabbits	16	Critical-size bone defects in calvaria	Histomorphometric	20 μg + peg + HA/TCP. 100 μg + peg + HA/TCP. Local.	Defect with no treatment; peg + HA/TCP.	51.1 ± 22.6%. 53.5 ± 22.7%	23.2 ± 10.1%. 34.3 ± 22.5%	TP + peg + HA/TCP increased the amount of bone regeneration compared with sites treated with peg + HA/TCP or empty control sites.
Jung et al., 2007 [17]	American foxhound dogs	6	Mandible	Histological, histomorphometry.	20 μg + peg + 350 μg/mL RGD. Local	Peg alone. Autogenous bone (positive control). Empty defect (negative control)	49.4 ± 7%	39.3 ± 5.7%. 50.5 ± 3.4%. 38.7 ± 1.9%	Synthetic RGD-modified PEG-containing TP is an effective system to achieve bone regeneration in bone defects in mandible.
Rowshan H. et al., 2010 [18]	Male Sprague Dawley rats	29	Mandible	Histological, radiographic densitometry	10 μg/kg/7 or 21 days. Subcutaneous.	10 μg/mL saline solution for 7 or 21 days. Subcutaneous.	n/a	n/a	A low dosage of TP administration might enhance the healing process in the early phase of a mandibular fracture model in rats.
Jensen S. et al., 2011 [19]	Male Göttingen minipigs	18	Mandible	Histological, histomorphometry.	20 μg + PEG + BCP. 20 μg/RGD + PEG + BCP. Local	Autogenous bone. BCP. PEG + BCP. EMD + PEG + BCP	35.54%. 37.89%	56.4%. 32.27%. 36.57%. 38.49%.	The study failed to demonstrate that EMD, TP, or TP + RGD, combined with experimental PEG hydrogel and BCP, stimulate bone formation.
Ali M. et al., 2012 [20]	Male Wistar rats	18	Mandible distraction	μCT at day 10, week 1, week 3	60 μg/kg, 3 times/week. Subcutaneous	Vehicle. (acetate buffer)	Day 10: 2.28 mm^3^ Week 1: 3.03 mm^3^ Week 3: 4.35 mm^3^	Day 10: 2.28 mm^3^ Week 1: 3.03 mm^3^ Week 3: 4.35 mm^3^	Intermittent TP boosted bone formation by 1.6–1.7 times, potentially reducing consolidation time.
Kuroshima et al., 2013 [21]	Sprague Dawley rats	80	Mandibular and maxillary extraction sockets	μCT, serum chemistry, histology	80 μg/kg daily, subcutaneous or intra-oral injection	Saline (vehicle control)	n/a	n/a	TP accelerates hard and soft tissue healing, preserves the alveolar ridge, and is equally effective via intra-oral or subcutaneous administration.
Kuroshima et al., 2013 [22]	Sprague Dawley rats	32	Maxillary first molar extraction sockets, grafted and nongrafted	μCT	Xenograft + 80 μg/kg/day, subcutaneous injection, 14 days post-extraction (some groups also 7 days pre-extraction)	Xenograft + saline (vehicle control)	n/a	n/a	Intermittent TP post-extraction significantly enhanced bone formation in both grafted and nongrafted sockets; pre-extraction PTH alone had no significant effect.
Tang et al., 2013 [23]	Japanese white rabbits	32	10 × 5 mm mandibular bone defect	X-ray, histology, histomorphometric, serum chemistry	25 μg/day/4 weeks subcutaneous	Saline (vehicle control)	n/a	n/a	Intermittent TP promoted mandibular defect healing by increasing osteoblast activity, enhancing bone turnover, and upregulating OPG.
Tsunori, 2015 [24]	Male Fischer rats	50	Critical-size bone defects in rat calvaria	Serum calcium concentration, alkaline phosphatase activity, micro-CT analysis, histological and histomorphometric analyses	15 μg/kg/day; 35 μg/kg/3 days/week; 105 μg/kg/day/week; 105 μg/kg/3 days/week Subcutaneous	Vehicle control; subcutaneous	40.3 ± 15.2% 48.9 ± 12.4% 55.3 ± 4.2% 66.2 ± 9.3%	10.3 ± 7.3%	TP groups showed greater and faster bone regeneration than controls in critical-size defects.
dos Santos et al., 2016 [25]	Wistar rats	45	Calvarial bone graft Histology fixed at mandibular angle	Histology, histomorphometric	2 μg/kg or 40 μg/Kg 3×/week for 30 days. Subcutaneous.	Saline (vehicle control)	76.42 ± 7.30% 80.75 ± 6.46%	80.14 ± 4.80%	High-dose TP preserved graft volume, prevented resorption, and increased bone mass; low dose had no significant effect.
Huh el al., 2017 [26]	Female New Zealand white rabbits	20	Sinus augmentation in healthy rabbits	Radiographic and histomorphometric analysis	10 μg/kg/5 days/2 weeks	10 μg saline solution/kg/5 days/2 weeks	10.85 ± 1.89%	10.45 ± 4.4%	Intermittent TP might not stimulate new bone formation in healthy rabbits during the first 4 weeks of healing.
Kajii et al., 2017 [27]	Male Wistar rats	18	Critical-size bone defects in rat calvaria	Micro-CT, radiographic analysis, histological and histometric analyses	0.1 μg/0.1 mL + ocp/col 1.0 μg/0.1 mL + ocp/col	ocp/col	29.2 ± 6.0%. 38.1 ± 7.1%	17.5% ± 3.6%	TP enhanced bone formation in a dose-dependent manner, confirming its osteoinductive effect with OCP/Col scaffolds
Göker et al., 2018 [28]	Female Wistar rats	90	Ovariectomy-induced calvaria defects	Measurement of bone turnover markers; dual-energy X-ray absorptiometry; microscopic observations	2.5 μg + px + mp; 2.5 μg + px; 2.5 μg + 15 mg sr + px; 2.5 μg + 15 mg sr + px + mp	Defects with no treatment; 1.5 mg sr + px;	n/a	n/a	Bone formation increased over time, with TP + px and TP + px + mp groups outperforming controls.
Zandi et al., 2019 [2]	Male Wistar rats	135	Autografted mandibular defects	Histology, bone healing scores, micro-CT	Iliac graft + 2 μg PTH/kg/day. Subcutaneous.	Negative control (no graft), control (iliac graft)	52.75 ± 2.83%	18.00 ± 1.87%. 38.50 ± 3.27%	The results showed that systemic administration of TP significantly enhances bone regeneration in mandibular defects treated with autografts.
de Oliveira et al., 2019 [1]	Male Wistar rats	78	Orchiectomy-induced alveolar bone modeling	Micro-CT, immunolabeling for RANKL/OPG, bone volume	0.5 μg/kg/day. Subcutaneous.	Orchiectomized without treatment; sham surgical procedures	54.96 ± 4.16%	41.23 ± 3.98%. 51.84 ± 3.29%.	Systemic TP enhanced alveolar bone modeling post-extraction in an androgen-deficient rat model.
Zandi et al., 2020 [29]	Albino Wistar rats	120	Unilateral mandibular fracture, surgically created	Histology	2 μg/kg/day. Subcutaneous.	Saline (vehicle control)	n/a	n/a	TP significantly accelerated fracture healing, showing more trabecular and mature bone at all time points at days 10, 20, and 30.
Dam et al., 2020 [30]	Female New Zealand white rabbits	20	Sinus grafting in ovariectomized rabbits	Bone mineral density (BMD), radiographic and histometric analysis	10 μg/kg/5 days/5 weeks. Subcutaneous.	Sterile saline solution in volume equivalent to the study group.	29.45 ± 6.04%	34.37 ± 5.33%	TP improved bone maturation, though not early volume, in estrogen-deficient sinus grafts.
Pelled et al., 2020 [31]	Yucatan minipigs	6	Segmental mandibulectomy in pigs	High-resolution X-ray imaging, biomechanical testing	1.75 μg/kg/day/8 weeks. Subcutaneous.	Sterile phosphate-buffered saline in volume equivalent to the study group.	46.3 ± 5.7%	32.3 ± 4.8%	TP significantly enhanced bone allograft integration and mechanical properties.
Emam etl al, 2020 [32]	Domestic pigs	6	Mineralized bone xenografts in mandibular defects	BMD in CBCT and micro-CT, nanoindentation, histology	20 μg + Bio-Oss. Local	Bio-Oss without PTH	n/a	n/a	Addition of TP to Bio-Oss improved mineralization, bone hardness, and regeneration.
Koca et al., 2021 [33]	Sprague Dawley rats	48	Critical-size defects in rat mandibles	Micro-CT, histomorphometry, osteoblast count	40 μg + allograft. Local	Empty defects; autograft; allograft	23.27 ± 0.15%	8.3 ± 0.25% 12.38 ± 0.16% 18.43 ± 0.16%	A single local dose of TP significantly improved allograft integration and bone healing.
Çamili et al., 2022 [34]	Male Wistar rats	20	Experimentally induced premaxillary expansion using helical springs	Histology, immunohistochemistry	60 μg/kg/7 days. Subcutaneous.	Saline injections, same volume	n/a	n/a	TP significantly enhanced bone formation at the midpalatal suture, increasing osteoblastic activity and upregulating osteonectin, osteocalcin, and VEGF
Wang et al., 2023 [35]	Male Sprague Dawley rats	70	Mandibular growth model	Morphometry, histology, immunohistochemistry PCR, immunofluorescence	8 mg/kg/4 weeks. 800 μg/kg/4 weeks. 80 μg/kg/4 weeks. Abaloparatide: 8 mg/kg/4 weeks. 800 μg/kg/4 weeks. 80 μg/kg/4 weeks. Subcutaneous.	Saline injections, same volume. Subcutaneous.	n/a	n/a	TP promotes site-specific mandibular growth, enhances chondrogenesis, reduces hypertrophy, synergistic with MA. ABL generally more potent, especially at condyle and angle; limited effect at anterior alveolar bone unless combined with MA.
Nakanishi-Kimura et al., 2024 [36]	Ovariectomized (OVX) and sham female Sprague Dawley rats	20	Mandibular bone (alveolar, buccal, lingual sites) and parietal bone	Histomorphometry, 3D confocal fluorescence analysis	30 μg/kg, 3×/4 weeks. Subcutaneous.	Saline injection (vehicle). Subcutaneous. OVX and sham-operated groups	n/a	n/a	TP induces site-specific perilacunar remodeling in mandibular alveolar bone, minimal effect in parietal bone; no net BV/TV change.
de Araújo et al., 2025 [37]	Female Wistar rats	30	Critical-size bone defects in rat calvaria	Histological, histomorphometry, immunohistochemical, and biomechanical	45S5 bioactive glass	Empty defect, 45S5 bioactive glass + 10 μg teriparatide	42.12 ± 5.34%	18.14 ± 2.25% 29.01 ± 3.68%	TP associated with bioactive glass, was successfully incorporated and had a positive effect on bone repair.

TP: teriparatide; VEG: vascular endothelial growth factor; BMP-2: bone morphogenetic protein; PDGF: platelet-derived growth factor; OCP/Col: octacalcium phosphate collagen; sr: stronim ranelate; px: poloxamer; mp: chitosan microparticles; micro-CT: computerized microtomography; RANKL: receptor activator of nuclear factor ligand; OPG: osteoprotegerin; BMD: bone mineral density; peg: polyethylene glicol; HA/TCP: hydroxyapatite and tricalcium phosphate; RGD: arginine–glycine–aspartic acid; EMD: enamel matrix derivative; MA: mandibular advancement; ABL: abaloparatide; OVX: ovariectomized; BV/TV: bone volume/tissue volume; n/a: not applicable.

## Abbreviations

The following abbreviations are used in this manuscript:

TP	teriparatide
GBR	guided bone regeneration
VEGF	vascular endothelial growth factor
BMP-2	bone morphogenetic proteins
PDGF	platelet-derived growth factors
OCP/Col	octacalcium phosphate collagen
sr	stronim ranelate
px	poloxamer
mp	chitosan microparticles
Micro-CT	computerized microtomography
RANKL	receptor activator of nuclear factor ligand
OPG	osteoprotegerin
BMD	bone mineral density
peg	polyethylene glycol
HA/TCP	hydroxyapatite and tricalcium phosphate
RGD	arginine–glycine–aspartic acid
EMD	enamel matrix derivative
MA	mandibular advancement
ABL	abaloparatide
OVX	ovariectomized
BV/TV	bone volume/tissue volume
